# Applications of Biomimicry in Architecture, Construction and Civil Engineering

**DOI:** 10.3390/biomimetics8020202

**Published:** 2023-05-15

**Authors:** Mariam AlAli, Yara Mattar, Mhd Amer Alzaim, Salwa Beheiry

**Affiliations:** 1 Engineering Systems Management, American University of Sharjah, Sharjah P.O. Box 26666, United Arab Emirates; 2 Department of Civil Engineering, American University of Sharjah, Sharjah P.O. Box 26666, United Arab Emirates

**Keywords:** sustainable development, biomimicry, architecture, building construction, civil engineering

## Abstract

Globally, the construction sector is regarded as one of the major contributors to greenhouse gas emissions, energy consumption, freshwater usage, resource utilization, and solid waste generation. With a constantly growing population and increased urbanization, this is only predicted to rise. Thus, achieving sustainable development in the construction sector has become an urgent necessity. The adoption of biomimicry in the construction sector is one of the most innovative concepts towards the shift into sustainable practices in construction. However, it has been noticed that the concept of biomimicry is very broad, relatively new, and abstract. Thus, after reviewing previously conducted research on the subject, a noticeable lack of knowledge on the effective implementation of the biomimicry concept was found to be present. Therefore, this research seeks to fill this knowledge gap whereby it aims to explore the development of the biomimicry concept in the field of architecture, building construction, and civil engineering with a systematic review approach of research related to biomimicry in these three fields. This aim is guided by an objective of developing a clear understanding of the application of the biomimicry concept in architecture, building construction, and civil engineering fields. The timeframe considered for this review is between 2000 and 2022. The qualitative exploratory nature of this research focuses on reviewing databases such as Science Direct, ProQuest, Google Scholar, and MDPI, as well as book chapters, editorials, and official websites to extract relevant information using an eligibility criterion of title and abstract review, inclusion of key terms, and detailed review of chosen articles. This study will improve the understanding of the biomimicry concept and how it can be applied in the built environment.

## 1. Introduction

The development history of planet Earth spans billions of years, and humans are a late addition to that long chain of evolution, with industrialization being the very last element. Despite the cited benefits of the industrial revolution, such as prolonging life and improving its different aspects, humans are still faced with the challenge of mitigating pollutions and environmental destruction as a result of the increased demand to create new products, hence threating the continuity of natural resources [1]. Accordingly, nature has been the biggest researcher throughout the history of life, as it has evolved on its own to solve several problems, such as the way animals and plants have efficiently utilized available resources and adapted to survive in harsh environments, which clearly shows that there is potential for imitating nature’s forms, systems, and processes to address the demanding challenges of today’s world [2]. Moreover, there is an urgent need to change the way buildings and cities are designed in the built environment in order to tackle climate change and biodiversity loss, in addition to the growing consumption per capita due to increased population growth and overall global urbanization [3].

In the eyes of designers and construction builders, nature’s different levels of complexity and forms have served as a basis of inspiration in the design and construction of buildings [4]. Moreover, a variety of questions have been raised by architects and designers as to how the complex relationship between nature and architecture can be harmonized to break away from the traditional design process that reviewed buildings as sheltering structures, as opposed to the full potential of modernizing its design and incorporating nature-inspired features to connect the natural world with the man-made world [5]. Hence, the term “Biomimicry” came into the picture, but how is it linked with the built environment, and how can it be interlinked with the built environment to serve as a problem-solving approach?

When looking at nature for inspiration, there are several terms that would surface. To further explain, Bio-inspiration is the use of nature to draw inspiration for problem solving, and within this broad term, Bio-inspired design (BID) emerges as a generic term that includes all the different approaches of design, which is inspired by life, nature, and living organisms. Moreover, there are three main terms for bio-inspired design, which are usually not distinguished from each other, and they are Bionics, Biomimetics, and Biomimicry [6]. Most scholars have agreed that the previously mentioned three terms are similar in terms of learning from nature with a focus on innovation, while noting that biomimicry differs from the remaining two in terms of emulating nature as a measure by focusing on preserving nature and life [7], therefore addressing the need to understand the functionality of the mimicked forms, not just emulating their structure for visual comfort. Other terminologies that may appear when researching biomimicry include Biomorphism/Biomorphic, Bionic(s), Eco-mimicry/Ecomimesis, and Organic Design, which are all under the umbrella of nature-based or bio-based design. To further illustrate, Biomorphic is about imitating the forms of living things; Bionic refers to copying or representing a function of a living thing to efficiently design technological or mechanically-driven systems; Ecomimicry deals with imitating nature on a larger scale, such as in the case of the design and socio-ecological management of communities and built environments; and Organic design involves the integration of symbolic associations, the use of biological principles allowing for nature-like relationships and harmony, and through the choice of biodegradable or reusable materials. In architecture, the use of biomimicry as a tool is referred to as biomimetic architecture [8]. Nevertheless, this article will explore biomimicry as the general umbrella, under which there are specific approaches such as ecomimicry or bio-morphism, which will be referenced where applicable.

In addition, Nature is considered as a knowledge source which provides some of the answers that humans need, as it has been evolving for more than 3.8 billion years through continuous trials, investigations, developments, and adaptation solutions, which all leads to the simple idea of being inspired by nature, or in other words, Biomimicry [9]. The term “Biomimicry” was first created in 1962, and originates from the Greek word “Bios”, meaning life, and “Mimesis”, meaning to imitate, or in other words “The conscious emulation of nature’s genius” as defined by Janine Benyus in 1997 [2]. Often the term Biomimicry is associated with the term “Biophilia”, which comes from Ancient Greek, combining bio (life) with philia (love) to symbolize the love of nature, and emphasizes the fact that humans are indeed drawn to the natural environment due to a connection between human psyche and natural systems, and similarly, biophilic design where buildings draw from the invited elements of the living world (such as plants or animals) as part of the urban design of building interiors and exteriors to influence a sense of belonging and well-being [10].

Moreover, biomimicry has been defined differently by researchers and scholars throughout the literature. For example, biomimicry is known in the sustainability field as “Mimicking the functional basis of biological forms, processes, and systems to produce sustainable solutions” [2]. Also, Biomimicry in Ecosystem Design Strategies revolves around “The emulation of strategies seen in the living world as a basis for design and innovation and has potential to contribute to the creation of more sustainable architecture and urban environments” [3]. Moreover, Biomimicry in design is defined as “An interdisciplinary approach, bringing together biologists, designers, engineers, among others, to study and transfer principles or mechanisms from nature to solve design challenges” [11]. As for Innovation and Infrastructure, Biomimicry is perceived as “Philosophy and interdisciplinary design approaches taking nature as a model to meet the challenges of sustainable development (social, environmental and economic)” [12]. Finally, Biomimicry in Building Design is acknowledged as “An ideology that combines biology with architecture to achieve a complete amalgamation of building and nature. It aims at studying the natural processes found in nature and uses it for the welfare of mankind” [13]. Accordingly, this article is mainly addressing the definition used in Building Design.

Within the context of the built environment, the application of biomimicry offers several benefits. Architectural researchers incorporated features from the environmental adaptation of organisms in the creation of sustainable buildings [14]. Furthermore, the emergence of energy-efficient buildings relies in its concept on the utilization of plant and animal survival mechanisms through their response to thermal stress and incorporates it by imitating fur or feathers in building insulation, as well as preventing excess heat loss from building extremities by using heat recovery systems mimicking the counter-current circulation in the legs of penguins and reindeers [4].

In order to better understand the process of biomimicry, it is important to identify the different approaches of biomimicry. Previous literature has identified several names for the two main types of biomimicry. First, there is the “Problem-based” approach where designers look at how organisms in the natural environment and ecosystems have solved their problems so as to introduce similar solutions, and it has also been named as “Design looking to biology”, “Problem Driven Biologically Inspired Design” and “Top-down Approach” [2]. However, even though this approach is based on identifying a problem through systematic steps utilizing the knowledge and skills of designers to come up with a common goal to create nature-inspired solutions [13], it still has the drawback of design limitations [15]. On the other hand, the second approach has been known as the “Solution-based approach”, “Biology influencing design”, “Bottom-Up Approach”, or “Solution-Driven Biologically Inspired Design”, which is based on the influence of biological knowledge on human design through extensive biological research prior to determining the design context [2]. To explain, this approach requires that a specific function or behavior of an organism or an ecosystem is identified first and then transformed to the design as a way of influencing it [13]. 

In terms of application, there are different levels of biomimicry that have been de-fined throughout the literature. For example, when a solution is needed to solve a design problem, biomimicry can be embodied in the analysis of different levels, such as form, process, and ecosystem [15]. Moreover, according to Pedersen Zari (2007), mimicry can have three levels: the organism (mimicking part of or a whole animal or plant organism), behavior (imitating a certain behavior or how an organism relates to the surrounding environment), and ecosystem (analyzing the overall interactions and functions), with further dimensions applied to each level, such as form (mimicking the appearance), material (the composition), process (how something works), construction (how something is made), and function (what something does) [16].

Therefore, when looking at the link between engineering and science, or more specifically, architecture and biomimicry, it is evident that designers can solve building design problems by looking at nature for solutions, which is further fueled by the main biomimicry dimensions defined by Benyus [17]. Nature as Mentor (valuing nature as a resource that has to be preserved instead of excessively extracting resources); Nature as Model (finding sustainable solutions for the design problem by modelling nature’s form, processes, systems, and strategies); and Nature as a Measure (considering nature’s quality control measures and evolution to establish an ecological standard for the sustainability of human’s innovations) [16].

Nonetheless, there is still a need to identify how biomimicry has been applied in fields other than architecture, which include building construction and civil engineering. These particular fields are essential in the built environment and constitute a major proportion of the construction projects that are carried out in different geographical areas around the world. Therefore, it is important to understand how nature has served as an inspiration in such fields and what applications, if any, are having a positive outcome in improving built environment aesthetics and providing beneficial functionality.

This paper aims at reviewing the literature addressing the evolution of biomimicry and its contributions in the built environment. This paper is further organized as follows: the Section 1 will address the literature review, followed by a brief explanation of the methodology of this research, proceeded by highlighting the main results and discussing the key findings related to applications of biomimicry in the built environment, and finally, the main conclusions and further recommendations.

## 2. Methodology

### 2.1. Data Collection

**Figure 1:**
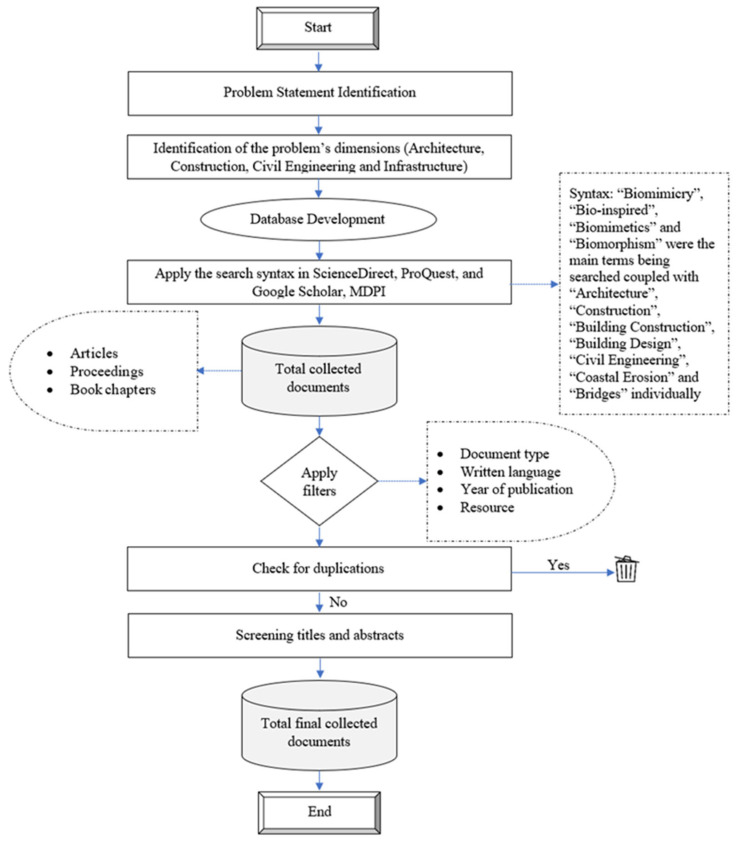
Detailed steps of the methodology.

This review was based on secondary research and investigated the concept of biomimicry from a large collection of scholarly articles found in the following databases: ScienceDirect, ProQuest, and Google Scholar (Figure 1). The reviewed publications spanned across the following fields: Architecture, Construction, Civil Engineering, and Infrastructure. Keywords used in the search included: “Biomimicry”, “Bio-inspired”, “Bio-mimetics”, and “Biomorphism” as the main term, coupled with “Architecture”, “Building Construction”, “Building Design” “Civil Engineering”, “Coastal Erosion”, “Road Construction”, “Bridge Construction”, “Bridge Engineering”, “Water Network”, “Sewage Network”, “Stormwater Network”, “Master Planning”, and “Transportation Engineering” through individual searches. The papers were selected based on the following search criterion: range of publication between years 2000 and 2022, relevance of title and abstract to the application of the biomimicry concept in one or more of the mentioned fields, and the content being strictly from an architectural or engineering aspect, while the exclusion criterion allowed for emitting any publication not belonging to a journal or a conference proceeding. However, due to the lack of scholarly articles discussing the application of biomimicry in the civil engineering/infrastructure field, scientific blogs and websites were referred to during the search through queries in Google Scholar.

### 2.2. Data Processing and Text Mining

For text mining and the review process of the selected papers, titles and abstracts were scanned to determine relevance to the chosen topics, followed by categorization of papers based on their specific fields. Themes and main ideas were extracted from the papers and recorded in tables using Microsoft Excel (Microsoft Office Home and Student 2019 version 2304, USA), and subsequently compiled to be added in the Section 3 of this review.

## 3. Results and Discussion

The primary search for papers through databases relied on using specific keywords relevant to the chosen fields of architecture, construction, civil engineering, and infrastructure (Table 1). Upon applying the selection criterion and implementing the inclusion and exclusion conditions, the resulting papers were sufficient to review the fields of architecture and construction, but due to a lack of academic papers relating to infrastructure applications, other resources were used such as blogs and websites.

### 3.1. Biomimicry in Architecture

Nature is a versatile source of inspiration that can help in developing the architectural design of traditional static and unresponsive buildings by introducing signs of life that allow those buildings to become contemporary in their concepts and exhibit features of sensitivity, adaptation, reactivity, and even evolutionary development [18]. Moreover, sustainable design solutions are considered a core element in sustainable development, since this refers to a source that is naturally energy efficient and also sustainable for billions of years, as biology can aid in enhancing beauty in architecture due to providing effective solutions from a source that is subject to the laws of physics and adopts the minimal consumption of energy necessary to maintain its survival [19].

There is a common thread between architecture and nature, observed in the shared attributes of growth and adaptation, and as such, most features of nature are translated into architectural designs in their geometrical form, without the necessary understanding of the structure and material logic, therefore resulting in a noticeable gap between the physical nature of the adopted form and the effects of its detailed structure that is seen in its natural environment, such as self-organization or complexity, but not necessarily reflected in the design concept [20].

Success stories of biomimetic designs have emerged on a global level since the seventies, as a result of the global oil crises in 1973 and 1979 and the need for global awareness, which has led discoveries such as self-cleaning hydrophobic surfaces relying on the Lotus Effect in 1976, and the development of the self-ventilation system of the Eastgate Building in 1996. However, in most of those cases, a biological push-up approach was adopted, where a certain property is observed or investigated as a natural resource from a scientific point of view and transferred to solve a technical problem [21]. On the other hand, the technology pull is considered a top-down approach where problems are solved by applying a biological concept or function, where it involves finding customized features and characteristics based on biomimicry [22]. Overall, the interests of architects, designers, and researchers did not have a proper understanding of the required tools or methods to apply the technology pull approach, but with the development of standards and methodologies aiding in the transfer of biomimetic features, the road was mapped for introducing environmental innovations in materials, facade systems, and buildings in addition to aiding in the development of urban and architectural regenerative projects that provide a positive impact on social, economic, and ecological systems [21].

According to Badarnah [23], natural systems for managing water have unique physical characteristics and occasionally use proactive measures to ensure effectiveness. In order to help identify viable mechanisms for use in building design, Badarnah presents a framework for water management tactics. The framework includes fundamental operations, associated procedures, and applied morphologies, with examples from nature to show how they are put into practice. Skin plays a significant role in water management in nature, resulting in the evolution of unique surface morphologies for condensation by creatures living in arid environments, which can be adapted to structures for moisture harvesting. Although not intricate, these morphologies have distinctive forms, sizes, and compositions. As a result, it is possible to create new systems with comparable functionalities by applying comparable physical principles [23]. In another study by Badarnah [24], an adjustable shading system modeled after leaves is shown. Plants have evolved special systems that allow them to choose the right amount of exposure for optimal photosynthesis in a given climate and location. The main determinants of light absorption in plants, where leaf distribution, orientation, and dynamics affect exposure to solar radiation, are physiology and geometry. A shading system that can track the spectrum of sun radiation throughout the day was created using principles and techniques that were taken from and derived from plants. With computer models that took azimuth and altitude angles into account, the efficiency of the shading system was examined during three crucial days throughout the year. As a result of the simulations, various area shading patterns were displayed. The distribution of the shading system’s components was determined by the interaction of azimuth and altitude angles. The device is flexible enough to rotate and reposition itself during the day, providing constant shading across the necessary plane at the envelope. Due to the system’s positioning normal to the sun’s rays, it is possible to accomplish the maximum amount of required darkened area with loose density of shade planes while avoiding self-shading or shading with high density of shadow planes for maximum energy gain [24]. In order to address and reduce the problems brought on by the use of evaporative cooling equipment in buildings, Zuazua-Ros [25] has outlined the definition, functionality, and uses of a bio-inspired heat dissipation system incorporated into buildings. Based on biomimetics, the approach uses dry surfaces rather than evaporation to simulate the heat dissipation mechanism used by some animals to cool themselves. By building a cooling panel prototype and analyzing it later, the original solution was produced. The data on the cooling power potential of the panel supports the use and further development of this system as a supplement to a cooling system and also validates it as a passive, renewable option. With many uses for its architectural integration, such as in emergency staircases or business center facades, this integration would significantly reduce consumption in buildings with a constant cooling requirement or in systems that contain cooling towers [25]. In another study, the growing issue of overheating buildings and their increased need for mechanical cooling has been addressed by examining the impact of surface texture on concrete panel heat loss capabilities using evaporative cooling. As the survival of organisms is maintained by utilizing morphological and behavioral means that complement physiological adaptation strategies to keep their body temperatures at very low ranges, this research developed a design solution through a biomimetic approach were the skin morphology of elephants was selected as a successful example that informed the conceptualization of a textured façade panel that employs evaporative cooling. The results of the systematic experimentation showed that the morphological variables of assembly and depth of texture had impacted heat loss, in addition to variable results in terms of impact of surface area to volume (SA:V) ratios on heat loss capabilities for different surface roughness textures. Accordingly, this study identified the potential utilization of morphological adaptations to buildings that can lead to reductions in energy consumption and less dependence on mechanical cooling systems by allowing buildings to cool passively [26]. Such research opens new prospects for novel thermal solutions for overheated buildings in warm temperature environments through bio-inspired solutions in the built environment and has the potential for expansion through developing guidelines for designers to develop applicable architectural thermal solutions inspired by nature for both existing and new buildings. Another study has also addressed the growing issue of increased amounts of sunlight entering into interior spaces in buildings that are designed with transparent materials such as glass paneling, which has an architectural allure. This research investigated kinetic façades as a way to provide suitable sunlight for interior spaces, integrated with a triple-identity DNA structure photosynthetic behavior, and a twist which was divided into a generation phase that used an evolutionary engine to produce potential strip patterns plus an evaluation for the kinetic façade using the Climate Studio software and Leadership in Energy and Environmental Design (LEED) version 4.1 criteria to validate daylight admission in an indoor space. By dividing the building envelope into four types, which were further assessed and compared in terms of receiving the daylight factor (DF) into the space with more or less sunlight, which resulted in a decreasing order of potential, as follows: entirely glass façade, twisting façade (the kinetic façade, version 2), rotating façade (the kinetic façade, version 1), and static façade. The result of daylight simulation showed that the newly designed kinetic façade (version 2) was able to filter beneficial daylight in indoor environments more efficiently than other building envelope types [27]. Such simulation studies are very beneficial in realizing the potential of applying nature-inspired solutions in the early stages of building architectural design to assess and resolve challenges that may otherwise not be seen until the actual operation of the building. 

### 3.2. Biomimicry in Building Structures

Biomimicry can be used to inspire new forms, processes, or systems. While biomimicry can impact the entire form and look of a building, it may also impact the layout of the mechanical systems, landscaping, structure, or façade. Building performance targets can also be guided by biomimicry. Nature can inspire building design concepts on various levels, whether for form, process, or system [11]. Green initiatives, particularly in the building sector, are becoming increasingly significant in recent years. Adoption and application of biomimetics as a basis in building projects is one of the most innovative techniques to achieve much more sustainable structures.

Essentially, adoption and application of the biomimicry concept in the building sector significantly minimizes the negative impact that a structure produces on the environment while ensuring improvement in quality of life for the people and animals living inside the building and surrounding it. It is an extremely promising form of modern design and construction, and already many of today’s most prominent developers and engineers are incorporating the biomimicry concept in their projects [28].

Since many years ago, nature has served as a significant source of inspiration for building and structural design, delivering the best models through continuous progress and optimization. This imitation of effective natural models seems to have been especially useful in the case of structural systems.

Mankind was inspired to create by a variety of examples from living and non-living organisms found in nature, including shells, crystals, spider webs, and soap bubbles. The three general applications of applied biomimicry and possible combinations of these are as follows [29]:

- Form—An idea would be making lightweight structures that resemble dragonfly wings.
- Processes —An idea would be simulating photosynthesis to capture solar energy.
- Systems—An idea would be constructing wall systems that replicate the homeostasis in organisms, which allows them to manage their interior conditions such as temperature.

Accordingly, a review of several examples on the application of biomimicry in building construction was performed to comprehend and analyze the various nature-inspired techniques used in building construction and how they have effectively responded to achieve efficiency and sustainability in building design. 

Biomimicry is about creating manmade systems that imitate the astonishingly efficient systems present in nature. The Eden Project [2,30,31] is the biggest greenhouse in the world which is located in a restored kaolinite mine. Since the site was still being mined throughout the design phase, they had to create a building that could be completed regardless of the ultimate ground levels. The result is a collection of varying-sized bubble-like domes strewn around the landscape. Inspired by nature, the engineers determined that employing geodesics (hexagons and pentagons) was the most effective technique to generate a spherical surface. These bubbles are comprised of a number of enormous hexagons that have been welded together and then inflated. Another example of bio-inspired structures is Sinosteel International Plaza. The goal for this building [32,33,34] was to construct a lightweight structure that used the least amount of material while yet passively regulating heat and allowing in enough light. The answer for the building’s design was to examine the ideal hexagonal honeycomb structure and include it in the design of the windows system. When it comes to the Eiffel Tower [33,35,36], the curving construction of the tower was supported by a lattice of studs and braces, similar to how the trabeculae support the curves in the head of the femur. Inspired by nature, a framework capable of efficiently sustaining a structure with an off-center load distribution was constructed. The Qatar Cacti Building [37,38] is a prime example of the biomimicry approach in structural engineering, with sun shades on the windows opening and closing in response to heat, just as the cactus transpires at night rather than during the day to retain water, thus mimicking the activity of the cactus, which transpires at night instead of during the day to retain water. The carbon neutral Utopian Village for Haiti, which is also called the Coral Reef Project [39,40,41], is inspired by a coral reef with fluid organic shapes as it is constructed on seismic piles to withstand seismic shocks from earthquakes which is similar to how coral reefs are basically underwater structural formations that are comprised of calcium carbonate as they are considered home to many marine animals and also help to balance the underwater ecosystem. One more example of bio-inspired structures is the Eastgate Centre [42,43], which is a shopping center and an office building located in Harare, Zimbabwe. The Eastgate Centre, largely made of concrete, has a ventilation system which operates in a similar way to termite mounds. On a behavioral level, the Rafflesia House [44] building was inspired by the Rafflesia flower’s natural mechanisms in achieving effective air conditioning and energy efficiency. The Swiss Re Headquarters in London [45,46], dubbed the “Gherkin,” is a 40-story skyscraper inspired by marine organisms known as “glass sponges.” These soak in water at the bottom and eject it at the top to filter nutrients; the ventilation system in the building replicates this movement. The Council House Building in Australia was the first to achieve the highest possible rating of six stars in Australia’s Green Star environmental accreditation program as it has the best sustainability features related to the biomimicry approach and linked to climate change mitigation and adaptation [15]. One last example of biomimetic applications in construction engineering is the Pearl River Tower in China [47,48], which is inspired by the structure of the sea sponge in achieving water and energy efficiency. Finally, applications of the previously mentioned biomimicry concepts used in building design to achieve sustainability in design are explained in detail in Table 2. These selected buildings were chosen based on their levels of sustainability and efficiency, their successful implementation of biomimicry in their design and construction, and their innovative design features that demonstrate a creative approach to biomimicry principles. Thus, these selected buildings have been extensively studied and have a wealth of information available on their design and performance. Representing a diverse range of building types and contexts, these selected buildings showcase the versatility of biomimicry in various applications.

Moreover, when looking at construction materials and considering the shift towards sustainable building practices, the use of bio-inspired materials has been observed through recent studies. For example, the use of sustainable building materials that incorporate natural polymers from renewable resources with good mechanical and biodegradable properties instead of natural cement has been achieved through developing sandstone building materials with gelatinized starch as a binder and sand as a filler. Conventional and natural coatings were used as a treatment to increase the hydrophobicity to reach the desired durability for industrial applications for waterproofing in building construction. The resulting artificial starch-based sandstones were classified as polymer-matrix composites with thermoplastic mechanical behavior. Additionally, the produced artificial starch-based sandstones reached a compressive strength of about 30 Megapascal (MPa) with a ratio of tensile strength to compressive strength of approximately 0.20 and low values of modulus of elasticity less than 2 Gigapascals (GPa) [49]. Such examples show that bio-inspired materials can potentially replace conventional building materials and be used in the construction industry. Moreover, plant-based Natural Fiber-Reinforced Polymer Composites (NFRP), alternatively named bio-composites, have been developed as a high-performance lightweight structure with topology optimized cross-sections. The conventional cross-sections of reinforced concrete in classic skeleton construction systems are often only partially loaded, which creates non-sustainable construction practices due to excessive material usage. Natural fibers were applied as a reinforcing agent within a bio-composite component using biomimetic topology-optimization scenarios. Furthermore, the developed morphology resembled a classic dendrological structure found in tree branches or root cross-sections and transformed it into new material-specific design expressions, and the fibrous morphology was defined by biomimetically-inspired orthotropic tectonics, generated with fiber path optimization software tools [50]. This example shows potential application of quick renewable resources in the building industry to reduce the construction ecological footprint by using bio-inspired sustainable building materials for future sustainable architectural practices and engineering design approaches. Another study designed and investigated a novel Helically Orientated Tubular (HOT) structure inspired by natural Bouligand-type energy absorbers. The Bouligand structure resembles plywood through a layered and rotated microstructure consisting of multiple lamellae, or layers, each one composed of aligned fibers. This study fabricated eight groups of HOT models with varying helix angles using 3D printing and evaluated their compressive properties through experiments and simulations. The results showed that the deformation behaviors and energy absorption mechanisms of HOT structures can be tuned by altering the helix angles. The auxetic HOT models exhibited an interesting behavior with negative Poisson’s ratios, while the hybrid and buckling HOT models showed higher energy absorption efficiency under relatively high compressive stresses and strains. This study demonstrates the adaptivity and flexibility of HOT structures under different loading conditions and reveals possibilities for optimal design with desirable energy absorption performance for various engineering applications [51].

### 3.3. Biomimicry in Civil Engineering

The construction industry is known as a main contributor for impacting the environment through factors such as pollution and high energy consumption, and the need to establish practices and strategies that embrace sustainability and a circular economy have become a necessity in order to minimize these impacts and to achieve social and economic benefits. The search for sustainable solutions to human challenges has reached a new level, impelling scientists, engineers, architects, designers, and innovators to refer to, and learn from, nature. Deviating from the traditional paradigm, the construction industry is slowly adopting the concept of biomimicry, from constructing energy efficient buildings to self-sustaining greenhouses [52].

Imitating nature through ecomimicry at an ecosystem level is a masterplan design in India. Situated between Mumbai and Pune in the Sahadyadri Mountains in India, Lavasa Hill Station is located near Mumbai and Pune, on the banks of a reservoir called Baji Palsalkar. It is the first hill city in India and has a total area of 25,000 acres at 300 feet above sea level. The Lavasa Hill Station area suffered deforestation caused by slash and burn practices. During monsoon season, heavy rains result in severe soil erosion followed by nine months of drought-like conditions causing huge volumes of water to easily evaporate. As a result, the water levels on the lake basin fluctuate by as much as nine meters seasonally [2]. To solve these problems by applying biomimicry, the engineers at HOK, a Global Design, Architecture, Engineering, and Planning Firm, designed buildings that mimic a tree’s taproot and circulation system to solve the scarcity of water during the drought season. To resolve the water overflow issue during the monsoon season, drainage systems were designed to mimic a harvester ant’s nest where ants construct their dams around their nest to redirect water away in multiple directions. The nest is usually 1–10 m in diameter and has tunnels descending 5 m or more and can usually be seen in dry and sandy to hard soils, with the nearby areas completely empty of vegetation. The engineers also designed roof tiles mimicking the “drip-tip” shape of the banyan fig tree to solve the water run-off issues as this design increases the water flow and creates friction that self-cleans the surface. Banyan tree leaves are described as large, glossy, leather, elliptical, and green in color, and like most figs, the leaf bud is covered by two large scales and as the leaf develops the scales abscise. Young banyan tree leaves typically have an attractive reddish tinge [53].

### 3.4. Biomimicry in Bridge Design

Inspiration from nature in designing bridges is not something new. Our ancestors have been building ancient bridges throughout human history after recognizing how nature uses structural forms to span physical obstacles. As a matter of fact, all basic types of bridge forms nowadays, such as beam, arch, and suspension, can find their ancient examples in nature. The only main difference between ancient peoples and our present civilization when it comes to building bridges is basically the materials used, as ancient civilizations used natural materials such as woods, vines, and stones, while we use steel and concrete [54]. Additionally, Hu et al. [55] suggest that there are five aspects of nature that can serve as inspiration for the development of bridge designs, namely: geometry, structures, mechanisms, energy, and intelligence. These five aspects have their corresponding goals that are inspired by nature. Geometry must have an eye-catching characteristic; Structures should be material-adapted; Mechanisms should be deployable and moving; Energy must be sustainable and multifunctional; and Intelligence requires that bridges should be smart and have self-control functions.

With the continuous search for technologies and innovations in design that promotes sustainability, engineers, designers, and architects look to natural design solutions for load-bearing structures such as bridges, as the designs of natural structures such as the spine of an animal or the branches of a tree can serve as an inspiration in designing bridges [56]. One example is the Forth Bridge in United Kingdom, which is located in Scotland and which is inspired by a Guadruped spine. It has a total length of 2467 m (m), including its approach viaducts, and is composed of three double cantilevers with two spans of 1700 feet (ft) suspended between them. Having been voted Scotland’s greatest man-made wonder back in 2016, the Forth Bridge is considered as a symbol of Scotland. Another example is The Musmeci Bridge in Italy that draws inspiration from seashells. This bridge is located above Basento River in Potenza City in Italy and is made of only one membrane of reinforced concreted of about 30 cm (cm) thickness and molded to form four contiguous arches. The bridge connects the city center of Potenza with the main access roads of the southern part of the city. A third example of biomorphism application in bridge design is The Tabiat Bridge in Iran, which mimics tree branches. The bridge is about 270 m in length which makes it the largest pedestrian bridge in the country of Iran. Situated in the north of Tehran, the bridge connects two public parks above one of the major highways of the city. The bridge consists of a three-dimensional truss and includes two continuous levels that sit on columns that mimic the shapes of trees. A fourth example is the Margaret Hunt Hill Bridge, USA, that mimics a spider web. Named after an heiress and philanthropist, the Margaret Hunt Hill Bridge spans the Trinity River and is situated in Dallas, Texas. This cable-stayed bridge has a total length of 368 m and has a main span of 184 m, which is supported by a steel arch that has a height of 122 m. The bridge’s platform is connected to the underside of the arch’s curved pylon by an array of twisting cables, and 58 white strands from the arch are connected and secured along the centerline of the platform. The 4.9 m diameter support is comprised of 25 individual segments and is secured with 9100 kg (kg) of bolts and an additional 480,233 kg of concrete. 

Recently, research inspired by the Beetle Elytra and the Buckling and Shear Properties of a Trabecular-Honeycomb Steel Web (THSW) offers a novel design for a box-girder bridge. This study uses the finite element technique (FEM) to examine and analyze the buckling stability and shear mechanism of THSW and recommends using η values of 0.2 or 0.25 for practical and economic reasons. THSW’s stress levels were found to be lower than a conventional corrugated steel web (CSW), but it still bore most of the shear force, resulting in an S-shaped deformation under shear loading. This is due to the fact that THSW’s design still follows the traditional assumption of web-bearing shear force and flanges-bearing bending moment, making it easy to promote and apply when under a shear load, as the THSW deforms consistently in an S-shape due to a synergistic shear mechanism that allows the trabeculae and honeycomb walls to yield in a sequential order, increasing the bridge’s ductility and load-carrying capacity. These findings lay the groundwork for further research and application of this bio-inspired bridge, which has the potential to improve the performance of conventional box-girder bridges with thin-walled steel webs [57].

Overall, the previous examples show that several bridges around the world have been designed and constructed based on mimicking the structures and forms found in nature through biomorphism. Furthermore, in order to create formidable and functional structures, it is important to take the best characteristics of natural structures and apply them in the design of bridges. For example, the flexibility and strength of spider webs is a well-known inspiration for tensile systems in some suspension structures, and the same goes for the branching system of trees, where its design is used as an inspiration for the load distribution in bridge support pillar design [56].

Moreover, adopting biomimicry through bionic designs does not always present a positive outcome. To explain, there are two cases of footbridges in Singapore, which are the Double Helix Bridge that draws its inspiration from the shape of DNA, and the Henderson Bridge that is patterned after natural waves. Both bridges have become landmarks in the community due to their visual impacts, however, partial mimicry from nature has led to a complicated design with a very high cost. The same scenario also applies to the Chunchua Bridge in China, which mimics the shape of a flower. The word Chunchua translates to Spring Flower in English, and this footbridge circles the intersection of Shennan and Nanshan Avenues in Shenzhen, China, and spans a space of 130 m long and 14 m wide, and has six staircases and thirteen elevators. Despite the evident use of this footbridge by pedestrians, it turned out to be a non-cost-effective design compared to traditional bridge designs. Finally, the design of the Peace Bridge in Calgary, Canada is inspired by fish bones. This pedestrian bridge is 126 m long and 8 m wide, with a total height of 5.85 m, and the structure of the bridge is inspired by fish bones. Nevertheless, this design drew flak from members of the public who said that the design was too modern for an old town known for its traditional structural designs. All the previous examples showed the need to evaluate the cost and applicability of bionic structures prior to constructing bridges in order to save both time and money and to satisfy residents’ needs and expectations.

### 3.5. Biomimicry in Coastal Protection

Another area in civil engineering that can benefit from being inspired by nature through ecomimicry is coastal protection. One example of applying biomimicry is the coastal protection that was conducted in the resort town of Blackpool on the United Kingdom’s northwest coast. The coast sea wall was damaged by continuous coastal dynamics and winter storms. By applying biomimicry as a solution, the high wall was replaced with a gently sloping set of steps stretching the length of the town, copying or mimicking the incline of sand dunes to dissipate wave energy. These steps acted as a mini wave wall to break the force of the waves incrementally [58]. The previous example clearly shows a successful application of biomimicry that resulted in protecting the coast of the resort town.

Another example of biomimicry being applied in coastal protection through ecomimicry is research conducted on the coastline of Florida, USA. A professor by the name of Keith Van de Riet along with a team of ecologists, biologists, engineers, and contractors designed and created concrete panels with root-like projections mimicking mangroves and installed it on the existing seawall. Mangrove reef walls are panels that enhance seawalls and create tidal habitat along waterfronts. These panels provide optimal conditions for a range of foundation species such as oysters, tunicates, sponges, and ecological producers that colonize the surface and enhance the constructed habitat. These mangrove reef panels create scaffolds for nature that enhances ecosystems. It also dissipates wave energy in the same way that natural mangroves do and improves the clarity of water by reducing suspended sediment and increasing the walls’ longevity by preventing sediment erosion. This technology was designed after the features of mangroves that help protect the coastlines of populated areas from soil erosion, as well as forming a defense from the impact of storm surges such as hurricanes. This also serves as a structure where natural organisms can thrive, as there were visible signs of colonization within only one and half years after installing the panels [59]. The previous application is also considered to have a positive impact in protecting the coastline of Florida, further showcasing the fact that applying the right form or structure from nature can have a favorable outcome in resolving challenges faced in the built environment.

An additional example on coastal protection and marine construction using biomimicry was conducted by a company from Israel that developed a technology called “ECOncrete”, which is a concrete material that serves as an alternative to the traditional ripraps used in stabilizing and securing shores in marine construction. ECOncrete technology designs concrete infrastructure that helps regenerate natural marine life and reduce ecological footprints whilst making infrastructures stronger to exceed international industry standards. As such, ECOncrete has a bio-enhancing agent that balances the concrete’s chemical composition; a texturing agent that allows organisms to thrive; and mold inserts which give added strength and durability. Moreover, this technology mimics natural marine surfaces from its chemical composition to its macro-features. Compared to traditional concrete structures, this technology encourages the growth of a variety of marine species from corals to oysters without sacrificing its structural integrity [60]. Additionally, one of many successful projects using this technology was the Brooklyn Bridge Park Project in New York, USA. To mimic natural rock pools, ECOncrete armor units were installed in the shore to help provide better shoreline stabilization, a wider tidal zone habitat, and an opportunity for public interaction with local marine life [61]. It is evident from the previous example that technologies such as ECOncrete have several positive impacts, including the preservation of marine species such as corals, oysters, and seaweed algae that help in rehabilitating habitats for fish, in addition to providing building blocks for the marine environment and offering coastal protection. Such examples show that a proper understanding of the functionality behind elements of structures found in nature can result in several beneficial outcomes for the environment.

Adding to the area of natural environment restoration, it is estimated that by around 2050 our planet will be home to approximately 9.3 billion people. Presently, planet earth is facing problems including food, water, and energy security, not to mention climate change, dwindling forests, and desertification. However, no problems are without solutions, but at the same time we cannot solve one problem at the expense of another. The Sahara Forest Project is one solution to what we are facing right now and possibly in the future if no action is taken. This project was established to achieve a goal, which is “Restorative Growth”, a goal that will create green jobs via production of food and freshwater, biomass, and clean energy. The Sahara Forest project facility locations are situated in low-lying, arid, and sunny areas that has little to no greenery and agricultural activity, hence the Pilot Facility was constructed in Qatar where a hot climate prevails the majority of the year. The Qatar Pilot Plant served as a platform for research to optimize and demonstrate environmental technologies that will enable restorative growth in desert areas globally. Following the success of the Pilot Facility in Qatar, the project established another facility in Jordan. Sitting on three hectares of land with two greenhouses and 3200 m^2^ of outdoor planting spaces, this facility can produce up to 130,000 kg of vegetables per year and up to 20,000 L (l) of fresh water per day [62]. Inspired by the Namibian beetle, the Sahara Forest Project is composed of various self-sufficient greenhouses which are similar to the beetle and capable of obtaining water by absorbing it from the air through a system of grilles and condensers placed on the roof. This innovation allows such dry areas deprived of resources to transform into biologically active and productive spaces which are abundant with vegetation and other forms of life [63]. From this example, it can be inferred that the desert can be transformed into a sustainable and profitable source of energy, water, food, and vegetation. It also shows an example of achieving circularity of resources by using the products or “waste” of one cycle as an input for another cycle. Accordingly, the previous case is a successful application of ecomimicry.

An additional example on preserving the natural environment is the Hamlet Waterfront Residential Development located in an urban area south of Ho Chi Minh City and adjacent to the Saigon River in Vietnam, where this 200-hectare residential development masterplan is based on an organic grid that is designed to mimic the existing environment and ecosystem. As the land in the development site is located in a floodplain which is used for agriculture, and as the site location is also crossed east to west and north to south with various small canals and tributaries leading to the Saigon River, this development will use the landscape greenery as a storm detention and infrastructure for water quality improvement, while using the canal network for storm water and flood management systems. The natural landscape and waterways will also serve as corridors for wildlife [64]. The previous example shows a successful implementation of emulating an ecosystem in design through the Ecomimicry/Ecomimesis process. 

### 3.6. Future Directions

It is evident that the transfer of knowledge from one discipline to another has great potential in the development of innovative solutions encountered through the usual intra-disciplinary methodological limits, where the collaboration between experts in the fields of biology, engineering, architecture, design, and even material sciences can aid in solving many obstacles through the introduction of nature-inspired features or native characteristics [65].

The application of biomimetic approaches to urban and architectural design is highly recommended, and has gained significant attention, as seen by the previous work of researchers. However, this particular field has not matured enough, and requires further enhancements through establishing a cross-linking between biological and engineering knowledge based on the development of educational programs to enhance the awareness of this vital integration. Additionally, joint databases could be established to gather all the necessary concepts, design strategies, and methodologies, as well as the successful biomimetic projects that have been carried out previously to be referred to as benchmarking practices for urban and architectural projects. Moreover, the role of digitalization should not be neglected as it can aid in the design of architectural structures based on an analysis of ecosystem elements and reproducing the form or function of an organism as an element in the built structure. Most importantly, life-cycle analysis should be incorporated as part of the evaluation methodology of biomimetic products or projects to determine their contributions to the environment, wither a positive addition or a negative impact, and their linkage with sustainable development and their impacts on biodiversity and ecosystems through the usage of proper indicators [66].

In terms of benefits, biomimicry does not contribute only to the aesthetic of a building or a structure; it contributes more importantly to the environment. With construction slowly adapting sustainable solutions such as biomimicry, the potential of lessening the negative impact of construction on the environment is limitless. In a study at the University of Johannesburg, South Africa, a group of respondents composed of biomimicry professionals, quantity surveyors, civil engineers, architects, and project managers with professional experiences ranging from 10–20 years were asked about the benefits of biomimicry implementation in the construction industry. As per the respondents, creating markets for green products and services tops the list of benefits of adapting biomimicry, followed by protecting biodiversity, conserving natural resources, restoring natural resources, and reducing global warming [67].

As a recommendation for future work, architects and designers are encouraged to consider investigating the optimal solutions inspired by nature at an early conceptual stage of any construction project to allow for proper decision-making based on the geometry of the chosen structures and to have a proper understanding of how certain functions found in nature could benefit the built environment, which can be achieved by performing advanced structural calculations and optimizations using mathematical equations to include both the topological transformation and the understanding of the structural logic [19].

## 4. Conclusions

Biomimicry is an innovative approach that can be applied to various fields, including architecture, building structures, civil engineering, bridge design, and coastal protection. Biomimicry draws inspiration from nature to create sustainable and efficient solutions to the challenges faced in the built environment. The use of biomimicry has proven to be successful in various architecture and construction projects, demonstrating the potential for solving technical problems and introducing environmental innovations in materials, facade systems, buildings, and marine constructions. By mimicking the efficient natural models, we can create manmade systems that have a positive impact on social, economic, and ecological systems, while minimizing the negative impact on the environment. The application of biomimicry in architecture and engineering should prioritize the use of sustainable materials, energy-efficient designs, and waste reduction strategies to minimize their environmental impact and move towards a more circular economy. In bridge design, designers and engineers should evaluate the practicality and cost-effectiveness of the design while incorporating biomimicry principles. Moreover, it is essential to consider the long-term sustainability and maintenance of these structures. The successful application of biomimicry in coastal protection and environmental restoration has demonstrated the potential to create sustainable and efficient solutions to the challenges faced in the built environment. It is essential to integrate biomimicry into the design and planning process of civil engineering projects, and further research should be conducted to develop new and innovative solutions inspired by nature. By doing so, a more sustainable and resilient built environment that supports the well-being of both humans and the planet can be created. 

Additionally, biomimicry has the potential to revolutionize the field of civil engineering by providing sustainable and innovative solutions to complex design problems. However, after exploring and analyzing databases of research and studies on the topic of biomimicry in civil engineering, it has been found that there is a gap in the literature with regards to this topic, as the concept of biomimicry in civil engineering is not widely or properly understood, especially in its core principles. This lack of understanding concerning its core principles has led to projects only partially adapting the principles of biomimicry and not as a whole. Despite its vast potential, biomimicry is not yet widely known or understood within the civil engineering community. The level of familiarity and knowledge about biomimicry between the architectural and civil engineering disciplines varies as well. While some engineers have started exploring biomimicry as a design approach, others are still skeptical or simply unaware of its benefits. Therefore, it is essential that the civil engineering community becomes more educated on the principles of biomimicry and its potential applications. This will require greater interdisciplinary collaboration and communication between architects, engineers, biologists, and other experts. In comparison to architectural design, civil engineering projects are often larger and more complex, with multiple stakeholders and significant impacts on the natural environment. Therefore, the adoption of biomimicry principles in civil engineering projects is even more crucial. The application of biomimicry in civil engineering projects can lead to more sustainable and efficient designs that reduce waste and energy consumption, enhance natural processes, and minimize environmental impacts. However, the improper understanding or implementation of biomimicry can also lead to unintended negative consequences. It is crucial that engineers approach biomimicry with a deeper understanding of the natural systems they are seeking to replicate, and ensure that their designs do not cause harm to the ecosystem. Additionally, there is a need for greater awareness of ethical considerations in biomimicry, such as the potential for biopiracy and exploitation of indigenous knowledge.

Biomimicry has enormous potential for transforming civil engineering, but its successful implementation will require a significant shift in the way engineers approach the design process. The civil engineering community needs to embrace interdisciplinary collaboration, incorporate biomimicry principles into their education and training, and adopt a holistic approach that considers both the technical and ethical implications of their designs. This paper has highlighted a significant gap in the literature concerning the application of biomimicry in civil engineering, particularly with respect to infrastructure such as bridges and roads. Despite the concept of biomimicry being well-defined and extensively utilized in the architectural field, its translation into civil engineering projects appears to be lagging. This is largely due to the fact that such projects are typically designed by civil engineers who may lack a comprehensive understanding of biomimicry principles and their potential application in the design of infrastructure. 

This discrepancy in the understanding and application of biomimicry between the fields of architecture and civil engineering is limiting the potential for more sustainable, efficient, and resilient infrastructural projects. It is crucial for civil engineers to gain a deeper understanding of biomimicry, and for the academic community to provide more resources and research in this area. This knowledge gap, if filled, could lead to infrastructural designs that not only mirror the efficiency and sustainability of natural systems, but also contribute to the resilience of the built environment. The lack of successful biomimicry implementations in civil engineering infrastructure projects underscores the urgent need for interdisciplinary collaboration, training, and research. Therefore, there is a significant opportunity for civil engineers, architects, and biomimicry experts to work together in creating a new paradigm for infrastructure design, one that is firmly rooted in the principles of biomimicry. This would not only help to address the current challenges in civil engineering but also pave the way for future advancements that could revolutionize the field. Therefore, it is imperative that further research and educational initiatives be undertaken to bridge this gap and fully harness the potential of biomimicry in civil engineering.

Throughout this journal article, several key challenges associated with the application of biomimicry in civil engineering projects were explored. The first major challenge is the difficulty of translating biological principles into engineering solutions. Nature’s mechanisms are complex, multi-functional, and operate on different scales, making it challenging to emulate these systems in man-made structures. Moreover, the properties of natural materials often depend on their structure at the microscopic level, which is difficult to replicate using current manufacturing techniques. The second challenge is the lack of interdisciplinary collaboration. Biomimicry requires a deep understanding of both biological systems and engineering principles. As such, successful implementation often requires collaboration between professionals from a variety of fields, including biology, materials science, architecture, and engineering. However, barriers such as differing terminologies, methodologies, and perspectives can impede effective interdisciplinary collaboration. The third challenge is the high initial cost associated with biomimetic materials and design strategies. Despite their potential long-term benefits in terms of reduced maintenance costs and improved sustainability, the upfront investment required for research, development, and implementation can be a significant barrier. This is particularly true in an industry that is often risk-averse and focused on short-term cost-effectiveness. The fourth challenge is the lack of regulatory support for biomimicry in civil engineering. Current building codes and regulations are typically based on traditional engineering approaches and may not accommodate or encourage the use of biomimetic strategies. This regulatory environment can make it difficult for innovative biomimetic projects to get approval, even when they offer significant advantages over conventional approaches. The fifth challenge is the need for education and training. Implementing biomimicry in civil engineering projects requires a new set of skills and knowledge, both about biological systems and how to translate these systems into engineering solutions. Current civil engineering education and training programs may not provide sufficient preparation for this task. Finally, the sixth challenge is the need for a cultural shift within the engineering profession. Biomimicry represents a fundamental departure from traditional engineering approaches, requiring engineers to think in terms of systems, processes, and materials that are inherently sustainable and resilient. This shift in mindset can be difficult to achieve and requires ongoing efforts to promote understanding and acceptance of biomimicry within the profession.

To conclude, while biomimicry holds immense promise for the future of civil engineering, its implementation is fraught with significant challenges. These include the technical difficulty of translating biological principles into engineering solutions, the need for interdisciplinary collaboration, the high initial costs associated with biomimicry, the lack of regulatory support, the need for specialized education and training, and the need for a cultural shift within the engineering profession. Addressing these challenges will require concerted efforts from researchers, educators, practitioners, policymakers, and the public. Despite these hurdles, the potential benefits of biomimicry in creating a more sustainable and resilient built environment are too significant to ignore. Furthermore, based on the findings and discussions presented in this article, the following recommendations are proposed for the successful integration of biomimicry in civil engineering projects:

- Fostering interdisciplinary collaboration: To fully leverage the potential of biomimicry in civil engineering, it is crucial to encourage collaboration among professionals from various fields, including biology, architecture, materials science, and engineering. This will facilitate the exchange of knowledge, ideas, and expertise, ultimately leading to innovative and sustainable solutions for civil engineering projects.
- Invest in research and development: Governments and private organizations should invest in research and development related to biomimicry in civil engineering. This will help expand the knowledge base, develop new biomimetic materials and design strategies, and refine existing technologies, leading to more efficient, sustainable, and resilient civil engineering projects.
- Implement policy support: Policymakers should create and implement regulations and incentives that promote the use of biomimetic strategies and materials in civil engineering projects. This could include tax incentives, subsidies, or preferential treatment for projects that incorporate biomimicry, as well as the development of industry standards and best practices.

## Figures and Tables

**Table 1:** Primary search outcomes.

Keywords	Database			
	ScienceDirect	ProQuest	Google Scholar	MDPI
“Biomimicry” and “Architecture”	11	3	N/A	4
“Biomimicry” and “Building Construction”	9	3	12	N/A
“Biomimicry” and “Civil Engineering”	1	1	8	N/A
“Biomimicry” and “Coastal Erosion”	2	0	11	N/A
“Biomimicry” and “Road Construction”	2	3	5	N/A
“Biomimicry” and “Bridge Construction”	2	0	5	N/A
“Biomimicry” and “Bridge Engineering”	3	0	5	N/A
“Biomimicry” and “Water Network”	0	0	0	N/A
“Biomimicry” and “Sewage Network”	0	1	1	N/A
“Biomimicry” and “Stormwater Network”	2	0	0	N/A
“Biomimicry” and “Master planning”	2	1	15	N/A
“Biomimicry” and “Transportation Engineering”	1	1	8	N/A
“Bio-inspired” and “Building Design”	4	2	8	N/A
“Biomimetics” and “Building Design”	3	1	9	N/A
“Biomorphism” and “Architecture”	9	19	15	45
“Biomorphism” and “Civil Engineering“	0	2	1	3
“Biomorphism” and “Building Construction“	0	3	1	2

**Table 2:** Applications of the Biomimicry concept in building design.

No.	Project Name	Inspiration	Biomimicry Concept	Added Value	References
1	The Eden Project, Cornwall	Soap bubbles and pollen grains	Air-filled geodesic hexagonal and pentagonal bubbles that contain hundreds of plants gathered from various temperatures and locations	The utilization of Green Tarriff Electricity, which is generated by one of Cornwall’s many wind turbines All of the enormous amounts of sanitized rainwater needed to produce the humid conditions	[2,30,31]
2	Sinosteel International Plaza	Beehive	The exterior is constructed of five varying sizes of hexagonal windows that multiply and grow across the building, generating an ever-changing image of the structure from every angle.	By mapping the diverse air flows and solar directions across the site, the honeycomb building enables the structure to be energy efficient. Energy efficiency achieved is 75%	[32,33,34]
3	Eiffel Tower	Thigh bone head	The outward flares at the base are reminiscent of the top curved part of the femur. Inner wrought iron braces strongly resemble the design of the original trabeculae within the femur.	Wind bending and shearing effects can be tolerated. The stable structure fixes the ventilation problem.	[33,35,36]
4	The Qatar Cacti Building	Cactus	Sunshades placed on the windows can be both opened and closed to accommodate the weather conditions and temperature, mimicking the function of the cactus, which transpires at night rather than throughout the day in order to maintain water.	Temperature Regulation Absorption and loss of heat controlled	[37,38]
5	Coral Reef Project Haiti	Coral reef	A self-sufficient energy village made of standardized and prefabricated parts to accommodate refugees from humanitarian disasters. Two duplex passive residences are interconnected by a transversal horizontal circulation that connects each unit. The entire project appears to be a large living structure made up of two waves dedicated to accommodating over a thousand Haitian families. Each residence’s roof represents an organic suspended garden.	For the local fauna and flora, this canyon represents a perfect tropical ecosystem. The roofs enable families to cultivate their own food and recycle their own waste. The project is eco-designed and incorporates all bioclimatic systems as well as renewable energy sources.	[39,40,41]
6	East Gate Center, Harare	Termite mound	The building opens and brings in more air, which is pushed up through ducts to support fans.	Energy reduction in HVAC by 100% Allows natural ventilation and lighting	[42,43]
7	Rafflesia House	Rafflesia flower	Concave and convex interior walls to control the current of air inside	Adoption of Net Zero Energy	[44]
8	Swiss Re Headquarters, London	Glass sponge	Utilizing triangulations on the outside such as those found on a glass sponge	Building energy consumption reduced to 80%	[45,46]
9	Council House Building in Australia	Termite mound	The structure uses a ventilation mechanism similar to that of a termite mound, employing natural convention ventilation stacks. The epidermis controls sunshine and brightness while maintaining a semi-closed microenvironment	Electricity consumption is decreased by 82% Gas consumption is decreased by 87% Water consumption is decreased by 72% Greenhouse gas emissions decreased by 87% Helped in isolating the indoor and outdoor environment Air is 100% filtered Providing shading and maximum daylight while maintaining visual comfort	[15]
10	Pearl River Tower, China	Sea sponge	Sponges draw their food by pumping thousands of gallons of water into their holes.	Wind turbines generate electricity by sucking wind into the tower’s four holes.	[47,48]

## Data Availability

Not Applicable.
